# Cancer and the Oral Cavity*Extracts from an address delivered to the Chicago Dental Society and published in full in the May *Dental Review*.

**Published:** 1917-05

**Authors:** Joseph C. Bloodgood


### CANCER AND THE ORAL CAVITY *

BY JOSEPH C. BLOODGOOD, M.D.

If we turn to the total number of deaths in 1914, we find 2,270 from the oral cavity and 1,967 from the skin. Why is the total number of cases in our skin clinics greater than the total number of cases of cancer of the mouth? It is due to the fact that cancer of the skin is recognized earlier and becomes more operable after a longer period of time. That is why we cure more cases of cancer of the skin. When I have the total number of cases of cancer in any locality and compare them with the total number of deaths, the group in which our results are worse, it corresponds with the group in the mortality statistics in which there are a greater number. For example, in cancer of the tongue, the total number of deaths is greater than the total number of cases of cancer of the lip, while cancer of the lip is a far more common form of cancer than that of the tongue, but our cures of cancer of the lip are greater than those from cancer of the tongue. They represent something like 15 to 16 per cent., 15 per cent. for the tongue and 60 per cent. for the fully developed cancer of the lip.

It is very interesting to have observed in the last few years that the mortality statistics really tell us what our operative results are in cancer.

In studying the relation of cancer to some etiologic factor, the investigation of 1,667 lesions of the mouth and

---

* Extracts from an address delivered to the Chicago Dental Society and published in full in the May *Dental Review.*

skin brings out a very important fact. Grouping them—mouth, face, scalp and the rest of the body—why is it the oral cavity ranks first with 638 cases? Because the oral cavity in the civilized individual is subjected to more chronic irritation than any other part of the external body. Our habits of eating, hot and cold, and the use of tobacco, are factors. Throwing out anything of theoretical consideration, the fact remains that cancer of the mouth is more frequent than cancer of the face. Why are cancers in the region of the face and scalp greater in number than cancers of the lower extremities and elsewhere, namely, 535 to 221? First, because the face of all parts of our body, the skin of the face has more congenital residues of cells than any other part of the body. That is one cause. The second is, the face is exposed to sunlight, while the rest of our body is pretty well covered, so that we have here in a large group of figures evidence that there is something else in the etiologic factor of cancer than Cohnheim's theory of a congenital residue, that is, chronic irritation.

When we study specifically, as far as we can, from our records, the 638 cases of cancer of the oral cavity, what one factor stands out first? I have read every single history myself that we have of these 638 cases; I have dictated and redictated them; I have written to the patients to tell me of the things they did not record. I have written to doctors of dead patients, and the country doctor, perhaps, knows a great deal more about his patients than the city doctor, and the one thing that stands out in these 638 cases of cancer of the mouth is neglected teeth. In the great majority of cases—I should say 60 per cent. of the cases—dentistry is conspicuous by its absence. I can not give you the exact figures, but very few of these cases have really been supervised by a good dentist. That is very important. It seems to me that is very essential evidence to give us hope that the problem of cancer of the mouth is the problem of education of the public in regard to cleanliness and good dentistry. I have written this, and I believe I have the evidence to support it, "Apparently it is as important to the health of the adult to keep the mouth clean and the teeth in good order as it is to the infant to be fed with clean, pasteurized milk." Now, if we could say that dentistry can prevent cancer of the mouth, it would be such a simple message that I doubt if we could get the public press to give it the same publicity as if I made the statement tonight that the juice of an orange would cure cancer.

The next prominent etiological factor—and I am not making a crusade against tobacco or alcohol, but it is evidence that we must consider—in cancer of the mouth is smoking. Perhaps it is not smoking alone. I got the impression that smokers, as a rule, are less cleanly in their mouths than those who do not smoke, and perhaps if the smokers had the same oral hygiene there would be less cancer, but the fact remains, nevertheless, that smoking is a very common factor, because why is it that cancer of the mouth is chiefly a disease of man, irrespective of whether you know they smoke or not? Why should cancer of the mouth be chiefly a disease of man? You may say men are less particular about their teeth than women. Among these 167 cases of lesions of the tongue, benign or malignant, there were but four women. Of course, as Hoffman, the great statistician says, you must have very large figures in order to eliminate errors, and that is not a very large figure, and I have not been able to get statistics from other clinics because they are not published in the same accurate way; but in these twenty-five years, in a clinic that gets most of its patients from a tobacco region, there were only four women, and three of these used snuff.

Among the 163 males, with cancer of the tongue or some other lesion, there is a statement in six histories that they did not use tobacco. Therefore, we have seen cancer of the tongue six times in men who did not use tobacco.

One of these men is living today, or the one that I know is living today. I saw him recently, and he admitted that he smoked up until two years when he came into the clinic, but when they asked him whether he had used tobacco, he said, "No," he was not using it then.

Among 117 cases of cancer of the oral cavity, confining it to the mucous membrane of the gum and mucous membrane of the cheek, and in few cases of the hard palate, that is, not of the tongue or floor of the mouth, there are eighteen women, and in none of these histories is it stated whether they used tobacco or not. So I can not say. Of the remaining ninety-nine men, in but three cases it is positively stated they did not use tobacco. In over seventy it is positively stated that they did use tobacco, and the young men who have been helping me in preparing this work told me by telephone as I left for Chicago that they had received six more letters stating they had heard from the individuals that they did use tobacco. To me that is a very important point as showing the importance of a positive statement. The statement as to whether they did or did not use tobacco can not be taken as evidence that they did not. I make this statement because Williams, who has written a splendid book on the Natural History of Cancer, states emphatically that his evidence showed that there is no relation between cancer of the mouth and tobacco.

Among the 276 cases of cancer of the lower lip the figures are about the same as for the tongue.

I want to leave the impression in your minds that in the past surgeons have exaggerated their operative cures for cancer, not purposely, but because they were anxious to show surgery would cure cancer, because if an individual waits for cancer to develop, in at least one-half of the cases it becomes inoperable. Of the operable cases, at the very best one-half are cured, so that the very best possible record has shown in the breast and lip 25 per cent. of cures. Recently we have increased our percentage of cures by placing among the cured cases of cancer lesions that are histologically doubtful, and I have submitted fifty specimens from such lesions to pathologists of the country from the breast in which there has been a difference of opinion as to whether they are cancer or not. The results in that group are 100 per cent. That group should be considered by itself.

I have simply shown what you can do with preexisting lesions, and I believe that cancer of the oral cavity in a large percentage of cases is a preventable disease. In a small percentage of cases it can be recognized in time to cure it without mutilation, and in a very small percentage of cases of these quiet, insidious cancers, I doubt if surgery will accomplish a cure, those that come under the head of osteomyelitis.

